# Informed Consent In Facial Photograph Publishing: A Cross-sectional Pilot Study To Determine The Effectiveness Of Deidentification Methods

**DOI:** 10.1177/15562646221075459

**Published:** 2022-01-24

**Authors:** Frank G Preston, Yanda Meng, Yalin Zheng, James Hsuan, Kevin J Hamill, Austin G McCormick

**Affiliations:** 1 Department of Eye and Vision Science, Institute of Life Course and Medical Sciences, 4591 University of Liverpool, Liverpool, UK; 2 Department of Ophthalmology, 4595 Aintree University Teaching hospital, Liverpool, UK

**Keywords:** deidentification methods, facial photographs, publishing ethics, patient confidentiality, patient anonymity

## Abstract

This study determined the effectiveness of three deidentification methods: use of a) a black box to obscure facial landmarks, b) a letterbox view to display restricted facial landmarks and c) a half letterbox view. Facial images of well-known celebrities were used to create a series of decreasingly deidentified images and displayed to participants in a structured interview session. 55.5% were recognised when all facial features were covered using a black box, leaving only the hair and neck exposed. The letterbox view proved more effective, reaching over 50% recognition only once the periorbital region, eyebrows, and forehead were visible. The half letterbox was the most effective, requiring the nose to be revealed before recognition reached over 50%, and should be the option of choice where appropriate. These findings provide valuable information for informed consent discussions, and we recommend consent to publish forms should stipulate the deidentification method that will be used.

## Introduction

Clinical facial photographs are valuable within the specialities of plastic surgery, ears, nose and throat (ENT), ophthalmology, oral and maxillo-facial surgery, dermatology, and dentistry, where outcomes are not always easily measured, but instead observed by the visualisation of aesthetic improvement (Bennett, Bonawitz, & Vercler, 2019). Clinical photographs are also utilised in research and educational purposes, to illustrate clinical findings, new surgical techniques, or postoperative results (Bhattacharya, 2014; Taylor, Foster, Dunkin, & Fitzgerald, 2008). The International Committee of Medical Journal Editors (ICMJE) state that photographs should not be published unless the patient provides written informed consent to allow publication if there is any doubt that complete anonymity has not been achieved (International Committee of Medical Journal Editors, 1995). Whilst the majority of patients consent to identifiable images being used for case notes, journals, and teaching, there is a strongly increased preference towards use of non-identifiable photographs in all modes of distribution (Lau, Schumacher, & Irwin, 2010; Roguljić, Peričić, et al., 2020). However, a paucity of research exists on the effectiveness of deidentification methods used to provide anonymity.

Medical journals, including those of the British Medical Journal (BMJ) and the Journal of the American Medical Association (JAMA), require journal specific consent forms to be completed by the patients before publication (BMJ, 2019; Koch & Larrabee, 2013). Both require the patient to confirm they have seen the photograph(s) to be included in the manuscript and have been given an opportunity to read it. However, numerous medical journals which publish facial photographs have been found to lack a policy on the publishing of these photographs (Roguljić, Buljan, et al., 2020). Nearly all journals publish online, some with open access, with articles promoted on Twitter and other social media platforms. Patients display lower acceptance rates for publication of both identifiable and non-identifiable images with websites, 40% and 73% respectively, compared to journals, 55% vs. 85% respectively (Lau et al., 2010). Differences between these modes of distribution are increasingly blurred. Patients may consent to the use of an image of themselves for publication in a journal without the knowledge that the article will appear online or disseminated across social media platforms. Due to this change from print-only publishing, there is a greater importance in discussing the potential reach of the photograph with the patient, and the effectiveness of any deidentification methods used.

Deidentification techniques such as masking, including the insertion of a black box over the eyes, nose or mouth, have been used historically in an attempt to conceal the identity of the patient within photographs (Koch & Larrabee, 2013). It has been argued previously that placing a black box across the eyes provides insufficient anonymity and simply ruins the photograph (Slue, 1989). This recognition has led to a consensus in the literature on the need to move away from deidentification techniques and gain consent (Bennett et al., 2019). However, studies have shown that eye masking has been used by journals to replace the need to obtain informed consent to publish facial photographs, while still claiming to comply with the Declaration of Helsinki regarding preserving the patient’s and subject’s rights (Shintani & Williams, 2012).

A previous study tested the utility of masking with a black box as a deidentification technique, concluding that some people remained identifiable irrespective of how much of the face was covered (Clover, Fitzpatrick, & Healy, 2010). However, there was no description of the methodology for black boxes generation or the areas of the face covered by each box. Other approaches, such as artificial facial composites have been shown to be effective, but are not practical to implement (Engelstad, McClellan, Jacko, & Melton, 2011). No studies have tested the effectiveness of the ‘letterbox view’ of the eyes and face, in which the facial photograph is edited by cropping out anything not within the region of interest. The effectiveness of the letterbox view applied to only one eye or side of the face alone, that we have termed a ‘half letterbox view’, has also not been investigated by previous studies.

The aim of this study was to determine the effectiveness of several deidentification methods for facial photographs. We investigated the use of a black box to obscure different facial landmarks, a letterbox view displaying limited facial landmarks, and a half letterbox view displaying limited facial landmarks of half the face.

## Participants and Methods

### Participants

The study was performed as a cross-sectional pilot study. Participants included were university students or staff. Student and staff participants were recruited through advertisement within the university department weekly email over a 4-week period. There were no exclusion criteria, and the educational level of participants was not assessed. All participants gave informed written consent to take part in the study and the data was collected and stored in accordance with the Data Protection Act (2018) (The National Archives, 2018).

### Celebrity Images

Facial images of 40 well-known celebrities were collated, using research by YouGov on ‘The most famous people in the UK’ to inform celebrity selection (YouGov, 2021). Celebrity faces were selected at a 50:50 male to female ratio and included a range of ethnicities. Portrait images of the celebrity looking straight at the camera were obtained via Google images, then formatted to 900    ×    900 pixels, edited such that the faces were of a similar size, and the backgrounds replaced with a solid black colour using Adobe Photoshop (Adobe Corp., San Jose, CA).

### Image Preparation of Deidentified Images

The standardised facial images were downsized to 256    ×    256 pixels to enable them to be put through a multi-task cascaded convolutional neural network (MT-CNN) (Zhang, Zhang, Li, & Qiao, 2016). The artificial intelligence (AI) algorithm utilised deep learning, identifying patterns from the raw data through the process of feature learning (LeCun, Bengio, & Hinton, 2015). The algorithm found areas of high and low contrast of the image intensity which corresponded to points of visual interest, enabling the generation of images with facial landmarks highlighted by standardised identifications (IDs) in the form of red dots (Zhang et al., 2016). This allowed standardised points to be generated and tailored for each of the different celebrity faces.

### Deidentification Method Generation Process and Image Dataset

**Figure 1.**
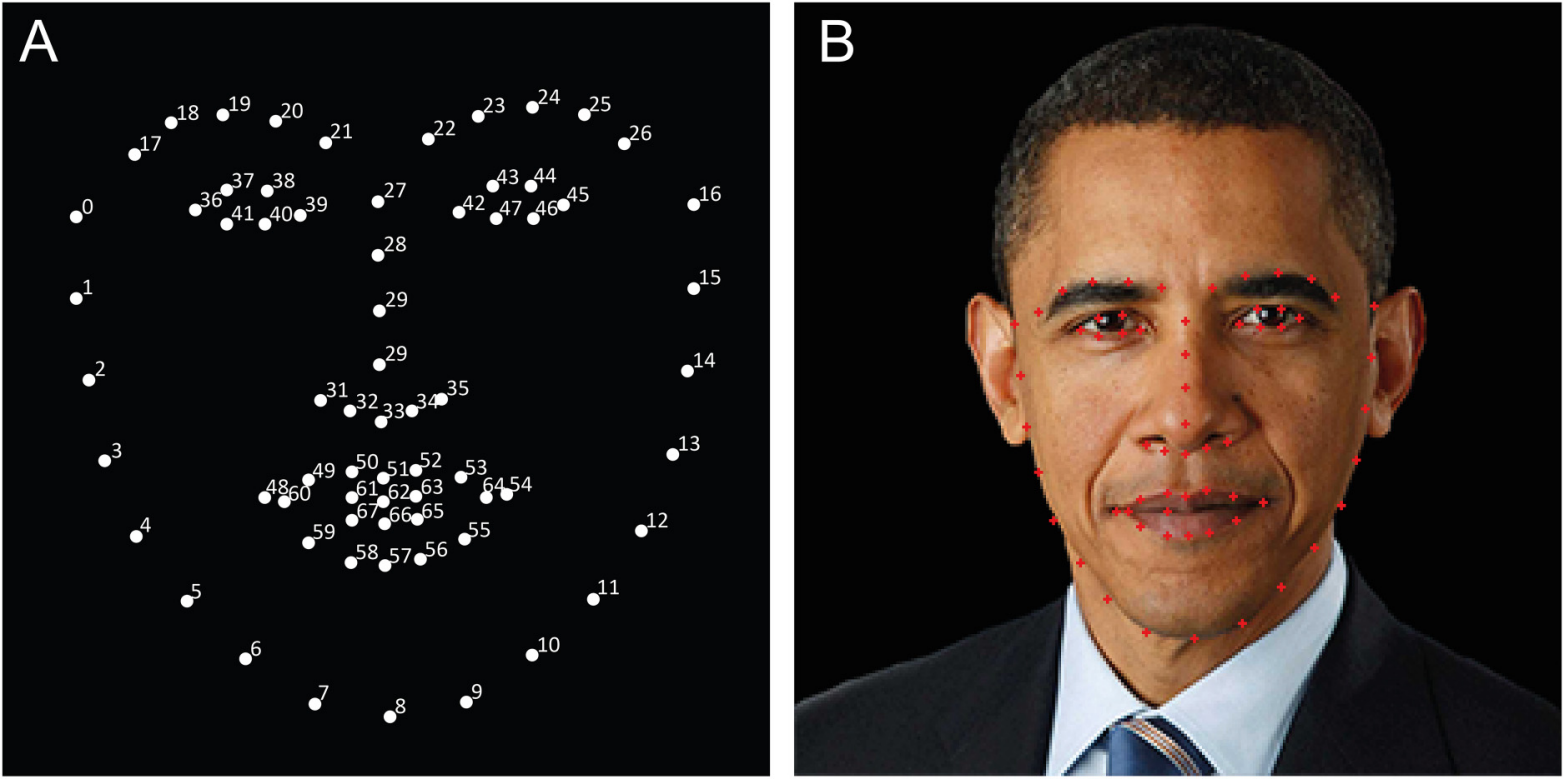
AI generated facial landmark identifications. (A) Facial landmarks identifications (ID) shown as numbered white dots. (B) Example facial image of Barack Obama demonstrating the corresponding facial landmark IDs shown as red dots.

The facial landmark IDs generated by the MT-CNN were used as guides in the manual generation of the deidentification methods for each image (Figure 1). As the IDs were specific to the individual faces, the deidentification methods generated were standardised but not uniform, instead being tied to the different sizes/proportions of each celebrity face and the features within. The deidentification methods were generated in Adobe Photoshop (Adobe Corp., San Jose, CA).

**Figure 2.**
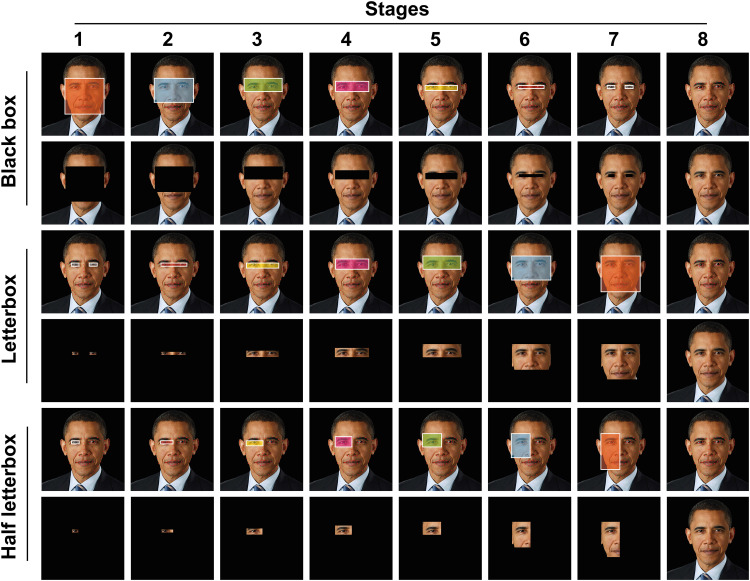
Area of face covered/revealed in each stage of the deidentification techniques with example images. Row 1, area of face covered in each stage of the black box deidentification method; row 2, examples of images used in the black box image group; rows 3, area of face revealed in each stage of the letterbox view deidentification method; row 4, examples of images used in the letterbox view image group; rows 5, area of face revealed in each stage of the half letterbox view deidentification method; row 6, examples of images used in the half letterbox image group.

Each deidentification method consisted of seven stages of deidentification before fully revealing the face on stage 8 (Figure 2). The black box deidentification method was generated by covering progressively smaller areas of the face with a black box according to the pre-determined stages (Table 1). The letterbox view deidentification method was generated by cropping the image to reveal progressively more areas of the face according to the pre-determined stages (Table 1). The half letterbox images were generated with the same method as the letterbox images but only displaying half of the face, with an equal number of right and left halves of the face used.

**Table 1.** Area of face covered/revealed by the deidentification methods

Stage	Black box (covered)	Letterbox view (revealed)
1	Peri-orbital region, forehead, nose, cheeks, and mouth	Two individual boxes encompassing each eye
2	Peri-orbital region, forehead, and nose	Each eye and the area in-between them
3	Peri-orbital region and forehead	Peri-orbital region without inclusion of the eyebrows
4	Peri-orbital region with the inclusion of the eyebrows	Peri-orbital region with the inclusion of the eyebrows
5	Peri-orbital region without inclusion of the eyebrows	Peri-orbital region and forehead
6	Each eye and the area in-between them	Peri-orbital region, forehead, and nose
7	Two individual boxes encompassing each eye	Peri-orbital region, forehead, nose, cheeks, and mouth
8	Whole face	Whole face

In total, the dataset consisted of 480 images. 8 images were generated for each deidentification method used on a celebrity face, with 20 celebrities being used in the black box image group, a further 20 different celebrities being used in the letterbox image group, and the same 20 then used in the half letterbox image group.

### Survey

PowerPoint (Microsoft Corp., Redmond, WA) slideshows were created containing the eight stages for each of the celebrity images used. Each image was placed on a separate slide in the sequential order. Surveys were carried out virtually over Zoom (Zoom Video Communications, San Jose, CA) or Microsoft TEAMs (Microsoft Corp., Redmond, WA). Examples of the different stages used within the black box, letterbox and half letterbox image groups were shown to the participants before showing them the test images. The participants were told by the investigator to inform them as soon as they could recognise the celebrity in the image, with either the name of the celebrity or an association. The first survey consisted of a PowerPoint slideshow containing the black box and letterbox image groups. The second survey consisted of a PowerPoint slideshow containing the half letterbox image group.

**Figure 3.**
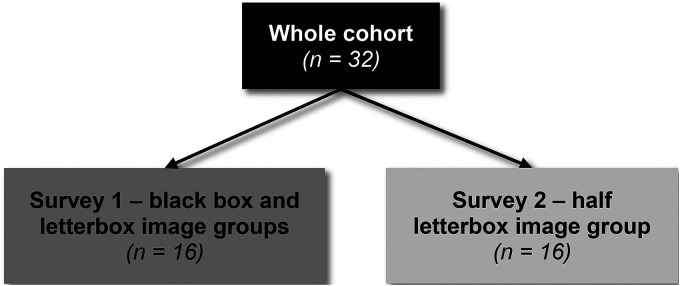
Flowchart of participants who completed each survey. n    =    number of participants.

We first compared black box and letterbox deidentification methods in a combined survey completed by 16 participants (Figure 3). We then sought to determine if the effect of using the letterbox method could be further improved by displaying only half of the face (half letterbox method). To do this we created a second survey containing the half letterbox image group. This image group contained the same 20 celebrities used in the letterbox image group from the first survey, but only displayed half the face. The second survey was completed by 16 new participants who had not participated in the initial study. The participant demographics were similar between the two surveys (Table 2).

**Table 2.** Demographics of deidentification survey participants

	**Black box and letterbox survey**	**Half letterbox survey**
*N*	16	16
Age (years)	27.1 ± 10.7	26.6 ± 8.0
Gender (female)	7 (44%)	8 (50%)

The main outcome measure was the stage at which the celebrity was first recognised, recorded by the investigator as a number between one and eight and stored in an Excel spreadsheet (Microsoft Corp., Redmond, WA). If the participant did not recognise the celebrity face once there was no deidentification, it was not used in the subsequent data analysis.

### Data Analysis

IBM SPSS Statistics 27.0.1.0 was used to perform all statical analysis on the data (SPSS Inc., Chicago, IL, USA). Descriptive statistics were performed on all the data collected. A 2-way ANOVA was conducted to test statistical differences between the identification stage at the end of the survey and the beginning of the survey to assess for a learning effect. A 1-way ANOVA Tukey’s post hoc was used to test if there was a statistical difference between the mean stage of recognition for each participant for each deidentification method. The statistical significance used for all analysis performed was p < 0.05.

## Results

Each image group contained 20 deidentified celebrity faces and was completed by 16 participants, resulting in 320 individual interactions with a celebrity face image per image group. Image identification stage of images at the end of the survey was not statistically different than the beginning of the survey, indicating there was no learning effect to increase identification ability. This allowed the data points for each face to be analysed independently (mean recognition stage for the first five celebrities versus last five: black box 1.3    ±    0.7 vs 1.8    ±    1.3, letterbox 3.9    ±    1.5 vs 4.2    ±    1.7 and half letterbox 5.6    ±    1.5 vs 5.0    ±    1.8, all p > 0.05 by 2-way ANOVA).

**Figure 4.**
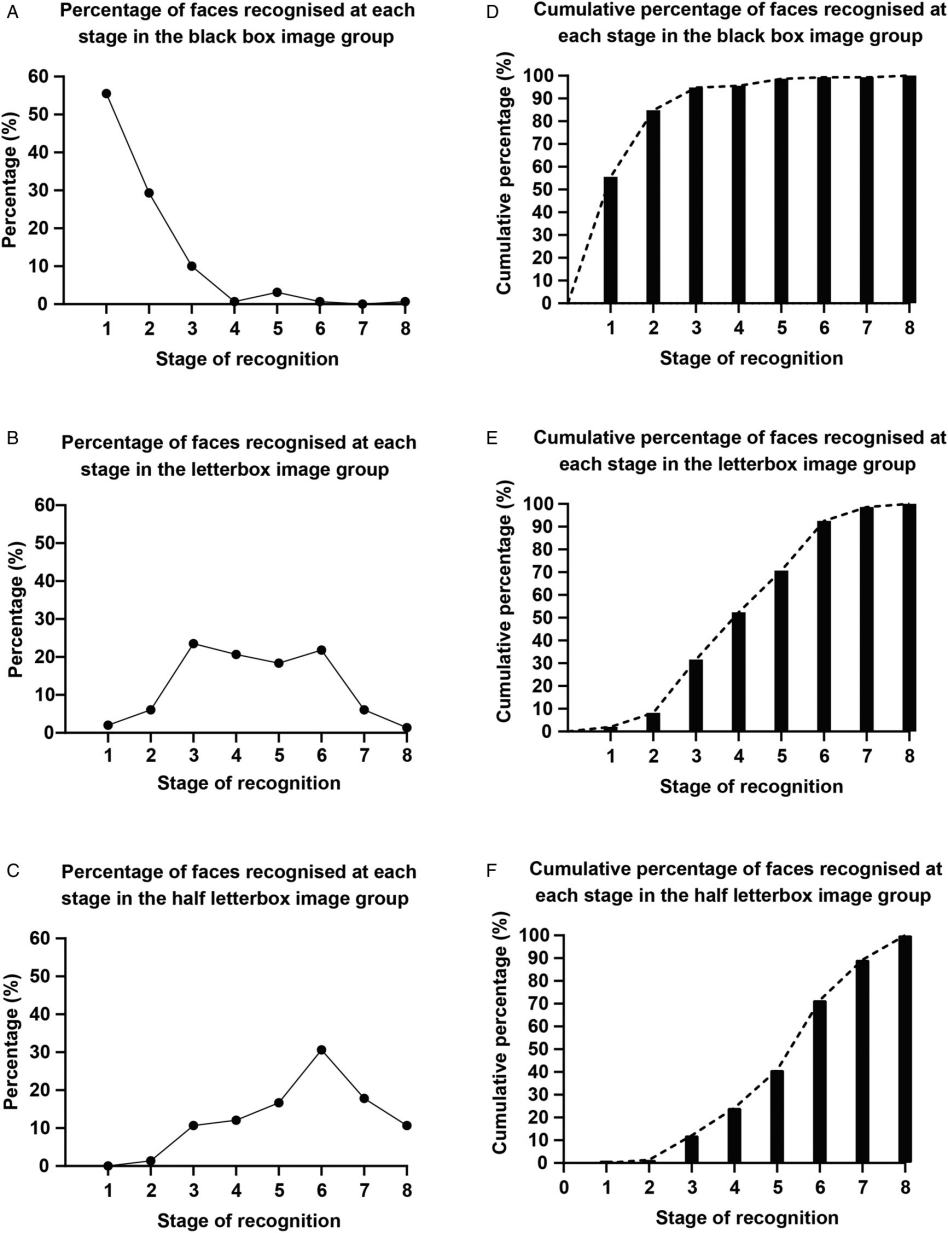
Recognition of faces using each deidentification method. Graphs displaying the percentage of faces first recognised at each stage in the black box image group (A); the letterbox image group (B); the half letterbox group (C); and the cumulative percentage of faces first recognised at each stage in the black box image group (D), letterbox image group (E), and the half letterbox image group.

In the black box image group (320 images), 30 images were not recognised even after full removal of all obscuring boxes, leaving a total of 290 data points for analysis (Figure 4. A, D). 55.5% of faces were recognised at the first stage with the black box covering the periorbital region, forehead, nose, mouth, and cheeks. After stage 2, which further revealed the mouth, the cumulative percentage recognised rose to 84.8%, and to 94.8% on stage 3, which revealed the nose. If the face had not been recognised after the first 3 stages, it was more likely that the person would not recognise the celebrity at all (30 images) than be able to recognise them at subsequent stages (15 images).

In the letterbox image group (320 images), 26 were not recognised upon full removal, leaving a total of 294 data points for analysis (Figure 4. B, E). Only 2% of faces were recognised at stage 1, which consisted of two individual boxes displaying each eye only. In stage 2, when the area between the two eyes was revealed, the cumulative percentage of faces recognised was 8.2%. The cumulative percentage of faces recognised after stage 3 was 31.6%, increasing to 52.4% after stage 4, when the eyebrows were included, 70.7% after stage 5 with more of the forehead being displayed, and 92.5% after stage 6 when the nose was revealed (Figure 2). If the face had not been recognised after the first 6 stages, it was more likely that the person would not recognise the celebrity (26 images) than be able to recognise them in subsequent stages (22 images).

In the half letterbox image group (320 images), 39 were not recognised, leaving a total of 294 data points for analysis (Figure 4. C, F). No faces were identified on stage 1, a single box encompassing one eye. It took until stage 6, revealing the nose, until most faces, 71.4%, were recognised, up from 40.9% on stage 5 when the peri-orbital region and forehead were displayed. If the face had not been recognised after the first 7 stages, it was more likely that the person would not recognise who the celebrity was (39 images) than be able to recognise them when the full face was revealed at stage 8 (30 images).

**Figure 5.**
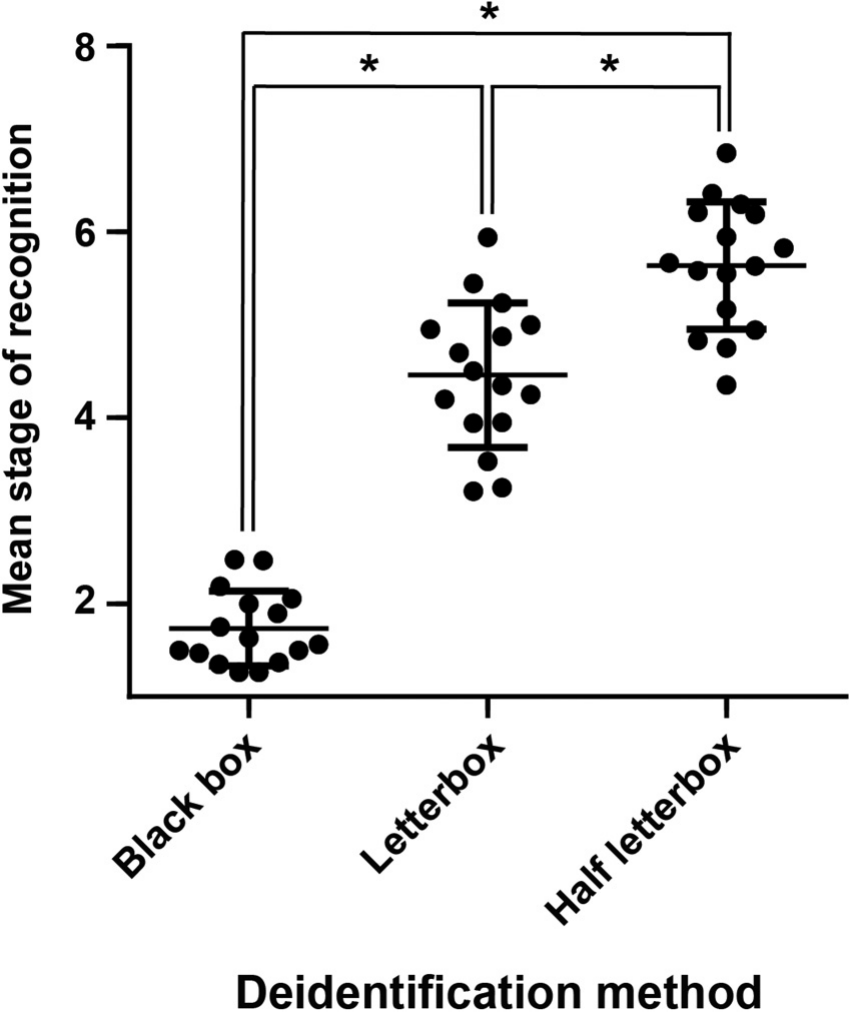
Deidentification method efficacy comparison. Dot plot of mean stage of recognition for black box, letterbox, or half letterbox deidentification techniques. Each point represents the mean score from one individual assessing 20 photographs. Line at mean, error bars standard deviation. * denotes p < 0.001 between groups by 1-way ANOVA with Tukey’s post hoc test.

Comparisons between the mean stage of recognition for each participant confirmed that the half letter box images were recognised later than the letterbox, and that both full and half letterbox were substantially superior to the black box method (mean recognition stage    +    SD, black box 1.7    +    0.4, letterbox 4.5    +    0.8, half letterbox 5.6    +    0.7, p < 0.001 for all comparisons 1-way ANOVA Tukey’s post hoc, Figure 5).

## Discussion

The results of this study indicate that a black box, even over the periorbital region, forehead, nose, mouth, and cheeks, cannot guarantee deidentification. This finding agrees with a previous study which looked at a T-shape black box over the eyes, nose and mouth (Clover et al., 2010). Some guidelines and papers have accepted a black box covering the periorbital region and eyebrows as a sufficient level of masking (Roberts, Troiano, & Spiegel, 2016; Shintani & Williams, 2012). However, at this stage, 95.5% of faces were recognisable. These findings, when combined with the reduced clinical quality and utility of the image, indicate the need to move away from this deidentification technique entirely. Although the letterbox view provides more effective deidentification than the black box method, it is still largely ineffective. More than half of the faces were recognised once the eyebrows were revealed, which concurs with previous studies indicating that eyebrows may be as influential in facial recognition as the eyes themselves (Sadr, Jarudi, & Sinha, 2003). The half letterbox view provided the greatest level of deidentification. Whilst less of the face is on display with the half letterbox method, hampering its clinical utility to display pathology, it would still be useful for some specialities for example ophthalmology and oculo-plastics, enabling unilateral eye pathologies to be displayed.

The question arises as to what level of anonymity is acceptable. Humans recognise faces holistically, as wholes as opposed to individual components and the relations between them (Farah, Wilson, Drain, & Tanaka, 1998). When parts of the face are artificially covered it cues the person viewing the face that the image has been altered, priming them to focus on the unaltered parts and mentally “fill in the blanks” (Engelstad et al., 2011; Tsao & Livingstone, 2008). Therefore, complete anonymity may not ever be possible. It has been argued that in situations where anonymity cannot be guaranteed, deidentification should be abandoned and consent gained (Koch & Larrabee, 2013). However, as an alternative, if the patient is willing to consent to publication of an image, the acceptable level of anonymity could be decided by the patient and included in the consent. As previous studies have found patients have a strongly increased preference towards use of non-identifiable photographs in all modes of distribution (Lau et al., 2010; Roguljić, Peričić, et al., 2020), the novel deidentification methods presented in this study require further testing to assess the acceptance for publication across all modes of distribution among patient stakeholders.

Consent forms, such as those used by the BMJ and JAMA (BMJ, 2019; Koch & Larrabee, 2013), could be adopted by all journals which publish clinical photographs of patients, especially those of the face. We propose that if a deidentification method is going to be utilised, the patient could confirm a discussion on the specific method has taken place. This would include stating the deidentification method and confirmation by the patient that the proposed effectiveness, or lack of research on the effectiveness, of the method had been discussed. In addition, due to the lower acceptance rates for publication of both identifiable and non-identifiable images on websites compared to journals (Lau et al., 2010), a greater emphasis is required in discussing the potential reach of the photograph with the patient. Inclusion in the consent form of the confirmation that a discussion on the potential reach the photograph may have has occurred with the patient would be one way to address this problem.

This study was limited to the use of well-known images of celebrities, with participants aware of this prior to completing the survey, meaning participants were considering a more restricted pool of individuals than would be the case normally. However, we deemed this is appropriate as it reflects the real-world situation of friends and family of a person with a particular medical problem having them in mind when viewing a medical photograph with the same medical problem. Another limitation included participants being allowed multiple guesses at the identity of the celebrity, meaning participants may have just had an inclination of the identity and not been completely sure. However, limiting participants to a single guess would not have worked with the method of progressively revealing more of the celebrity’s face, as participants who would have recognised a celebrity at a later stage would have been recorded as ‘not recognised’ for an incorrect answer at an earlier stage. The generation of facial landmarks using an AI algorithm is a strength of this paper by ensuring standardised identifications tailored to each celebrity face to account for differences in facial size and feature proportions. However, the subsequent manual generation of the deidentification methods may have led to minor differences between celebrities. A superior method would have been to produce the deidentified images using a further AI algorithm using the landmark identifications generated by the current AI algorithm. A further limitation was the small number of participants recruited.

Overall, this study argues that deidentification methods should not be used in place of consent, but provides the information required to inform consent. We recommend that specific consent forms for publication could include information on the deidentification method that will be used and that the effectiveness of that method has been discussed.

## Best Practices

- Informed consent should be sought and gained for the publication of images of patients/research participants.
- Patient consent forms for publication could be adopted by all journals which publish identifiable photographs.
- If a deidentification method is going to be used, the journal’s patient consent form could stipulate the deidentification method and confirm the proposed effectiveness has been discussed.

## Research Agenda

Further research is required to test the effectiveness of deidentification methods not looked at this paper. Examples include different forms of masking such as blurring, cloning neighbouring skin, and coarse pixelation (Roberts et al., 2016). This information is required to enable patients to be counselled about the proposed effectiveness of these techniques, to enable informed consent to be gained. Additionally, the acceptance for publication across all modes of distribution needs to be determined among patients and research participants.

## Educational Implications

The study provides information relevant to all authors who wish to publish facial photographs. It allows them and their subjects to understand the effectiveness of the different deidentification methods. This is a pre-requisite to them making an informed choice and giving meaningful consent. Implementation of patient consent forms, which allow the deidentification method to be stated and enable confirmation that the effectiveness has been discussed, would need to occur via individual journal editorial staff.

## Data Availability

The data is publicly available upon request with restrictions in accordance with ethics approvals. Data access request to the remaining data should be made to AM (austin.mccormick@liverpoolft.nhs.uk).
